# Omalizumab controls surface phenotypes of dendritic cells and monocytes in asthma

**DOI:** 10.1016/j.jacig.2025.100523

**Published:** 2025-06-23

**Authors:** Agnes Yang, Laurie Baert, Katherine Upchurch, Mark Millard, Matthew Wiest, Chao Gu, HyeMee Joo, SangKon Oh

**Affiliations:** a Department of Immunology, Mayo Clinic, Scottsdale, Ariz; b Institute for Biomedical Studies, Baylor University, Waco, Tex; c Martha Foster Lung Care Center, Baylor University Medical Center, Dallas, Tex

**Keywords:** Asthma, IgE, omalizumab, dendritic cells, monocytes, CD88

## Abstract

**Background:**

Omalizumab can provide clinical benefits to a fraction of moderate-to-severe asthma patients. However, the mechanisms of action of omalizumab have not been fully understood.

**Objective:**

This study investigated whether omalizumab could affect the frequency and surface phenotypes of blood circulating dendritic cell (DC) and monocyte subsets, which could be associated with the mechanisms of action of omalizumab.

**Methods:**

Longitudinal analyses of the frequency and surface phenotypes of DC and monocyte subsets in fresh whole blood of moderate-to-severe asthma patients (n = 45, 34 with response and 11 without response, from baseline to week 26) were performed by flow cytometry. Nonasthmatic subjects (n = 22) were also used as controls at baseline.

**Results:**

Omalizumab decreased myeloid DC/plasmacytoid DC ratio by increasing the frequency of plasmacytoid DCs. In addition to the decrease of surface FcεRI expression, omalizumab also downregulated HLA-DR, CCR7, and costimulatory molecule expression on both myeloid DCs and plasmacytoid DCs. Omalizumab also decreased CCR7 and HLA-DR expression by monocyte subsets. Omalizumab-mediated reduction of surface CD88 expression on monocytes was associated with asthma symptoms.

**Conclusion:**

This study provides new insight into omalizumab’s mechanisms of action. Data from this study will help us understand the roles of serum IgE in shaping surface phenotypes of DCs and monocytes in asthma patients.

Although the pathophysiology of asthma remains to be fully understood, IgE binding to a high-affinity IgE receptor (FcεRI) expressed on basophils, mast cells, and antigen-presenting cells (APCs), including dendritic cells (DCs), is one of the major processes leading to the pathogenesis of asthma.^1^^,^^2^ Omalizumab, a neutralizing anti-IgE antibody, can thus provide asthma patients with improved clinical benefits. However, 17% to 35% of moderate-to-severe asthma does not respond to omalizumab therapy.^2^, ^3^, ^4^, ^5^, ^6^ It is important to understand the mechanisms of action of omalizumab, which will eventually help us design novel therapeutics that can provide improved care to a wider range of asthma patients.

Omalizumab binds to the Cε3 domain of free IgE, resulting in the prevention of IgE’s binding to Fc epsilon receptor I (FcεRI).^7^^,^^8^ Omalizumab does not bind to FcεRI- or CD23 (FcεRII)-bound IgE, which could cross-link receptor-bound IgE.^4^ Instead, omalizumab downregulates surface expression of FcεRI on mast cells, basophils, and APCs by neutralizing free IgE in patient sera,^9^, ^10^, ^11^ as IgE-free FcεRI is unstable and quickly internalized and degraded.^12^^,^^13^ FcεRI expression levels thus correlate with serum IgE levels.^14^ Therefore, omalizumab can prevent IgE-mediated activation of mast cells and basophils, leading to reduced degranulation and secretion of proinflammatory mediators.^15^, ^16^, ^17^, ^18^, ^19^ Decreased expression of FcεRI on APCs^9^^,^^10^ can also limit IgE-bound allergen uptake followed by a decrease of allergen-specific inflammatory response.

DCs are the major APCs that can induce and control inflammatory responses.^20^^,^^21^ In humans, there are two major subsets of blood circulating DCs: myeloid DCs (mDCs; Lin^−^HLA-DR^+^CD11c^+^CD123^−^) and plasmacytoid DCs (pDCs; Lin^−^HLA-DR^+^CD11c^−^CD123^+^). Both mDCs and pDCs can induce and activate antigen-specific T-cell response, but pDCs also play critical roles in antiviral defense via rapid type I interferon production.^22^^,^^23^ It is also known that both mDCs and pDCs can contribute to allergic inflammatory T-cell responses, which can eventually lead to the enhancement of IgE production in B cells.^24^, ^25^, ^26^, ^27^ Of interest, pDCs can also alleviate allergic inflammatory response.^28^^,^^29^ Teach et al^30^ also reported that omalizumab improved IFN-α responses to rhinovirus, and greater IFN-α increases are associated with fewer asthma exacerbations in inner-city asthmatic children.

To induce and activate allergen-specific inflammatory T-cell response, DCs need to be activated and matured. Once DCs have matured, they express increased levels of HLA-DR, C-C chemokine receptor 7 (CCR7), and costimulatory molecules, including CD80, CD83, and CD86, which contribute to T-cell activation.^31^ CCR7 on DC surface facilitates their migration to lymph nodes.^31^^,^^32^ A previous study have also reported elevated levels of CD80 and CD86 in patients with asthma.^33^

Although DCs express an αγ2 trimer form of FcεRI, which lacks the signal-enhancing β-chain found in the tetrameric FcεRI on basophils and mast cells,^34^^,^^35^ IgE binding to the FcεRI on DCs can still induce intracellular signals. This suggests that serum IgE can contribute to the enhancement of allergic inflammatory response, not only by the promotion of allergen uptake via FcεRI^36^^,^^37^ but also by shaping the phenotypes of FcεRI-expressing APCs—a result that to our knowledge has not been previously reported. Neutralizing serum IgE with omalizumab could thus potentially modify surface phenotypes of APCs, including DCs and monocytes, making them less inflammatory. In addition, omalizumab-induced surface phenotype changes on DCs and monocytes might differ between omalizumab response and nonresponse.

To address the above questions, longitudinal analyses were conducted on the frequency and surface phenotypes of DC subsets (mDCs, pDCs), and 3 different CD14^+^ monocyte subsets (CD14^+^CD16^−^, CD14^+^CD16^+^, CD14^dim^CD16^+^) using fresh whole blood collected from both those with response to omalizumab (n = 34) and those without (n = 11). Data from this study demonstrate that omalizumab can modulate surface phenotypes of DCs and monocytes in asthma patients by reducing their inflammatory properties.

## Methods

### Study subjects

Under the protocol approved by the institutional review board, 45 moderate-to-severe adult asthma patients receiving treatment with inhaled corticosteroids and/or long acting β_2_-adrenoceptor agonists were recruited.

Disease severity was defined by a combination of low Asthma Control Test (ACT) score (<19), low lung function (defined as forced expiratory volume in 1 second score of <80% of predicted), and frequency of symptoms, including total number of days with symptoms per week and nighttime sleep disruption of more than once per week.

Patients who were pregnant, who were under the age of 18, and/or who had recently received omalizumab therapy were not included in this study. Age- and sex-matched 22 nonasthmatic control subjects were also enrolled under the same protocol.

All experiments were performed in accordance with the Declaration of Helsinki. The characteristics of patients and nonasthmatic control subjects have been previously reported (see Table E1 in the Online Repository available at www.jaci-global.org).^5^

### Study design

The design of this study has been previously described.^5^ In brief, patients were prescribed omalizumab (Xolair, provided by Genentech) according to their physician’s recommendation, dosed as per the manufacturer’s dosing table according to the patient’s serum IgE and body weight. All patients (n = 45) had asthma that was uncontrolled despite treatment with inhaled corticosteroids and/or long-acting β-agonists. Blood was collected 1 week before treatment initiation and again on the first day of treatment before receiving omalizumab as baseline samples. Once beginning treatment, blood samples were collected at week 6, week 14, and week 26 after treatment initiation.

At each blood draw, patients filled out the ACT questionnaire and an evaluation form for the total frequency of symptoms, inhaled steroid/β-agonist receipt, and nighttime awakenings per week. A lung spirometer test was also performed.

Patients with and without disease that responded to therapy were classified as follows. Response to omalizumab was according to improvements in asthma control; those without response still exhibited uncontrolled asthma.^6^^,^^38^, ^39^, ^40^, ^41^ Uncontrolled asthma (nonresponse group) was defined by an ACT score of <19 with asthma symptoms, inhaled steroid/β-agonist receipt at least twice a week, nighttime awakenings at least once a week, lack of change in asthma control medication, and other indications of inadequate asthma improvement confirmed by physicians, similar to other studies analyzing omalizumab response.^38^, ^39^, ^40^, ^41^ Because lung function (measured as forced expiratory volume in 1 second) has not been shown to be a reliable indicator of response to omalizumab,^42^ it was not included in our response classification.

### Whole blood staining

Whole blood (200 μL) was stained with the indicated antibodies, and 50 μL per well of brilliant stain buffer (Becton Dickinson [BD], San Diego, Calif) for brilliant violet fluorochrome stability was added. Three subsets of DCs, mDCs (Lin^−^HLA-DR^+^CD123^−^CD11c^+^), CD141^+^ mDCs (Lin^−^HLA-DR^+^CD123^−^CD11c^+^CD141^+^), and pDCs (Lin^−^HLA-DR^+^CD123^+^CD11c^−^) and 3 subsets of monocytes (CD14^+^CD16^−^, CD14^+^CD16^+^, CD14^dim^CD16^+^) were examined. Blood was lysed and cells were fixed with BD lysing solution. Stained cells were analyzed on an LSR Fortessa flow cytometer (BD), and the results were analyzed by FlowJo software (Becton Dickinson, Franklin Lakes, NJ). Detailed information for the antibodies we used in this study is summarized in Table E2 in the Online Repository available at www.jaci-global.org. To count cell numbers, 20 μL of CountBright absolute counting beads (Life Technologies; Thermo Fisher Scientific, Waltham, Mass) was added to each well. Cell counts (determined as number per microliter) were calculated by using number of cell events *(A)* divided by number of bead events *(B),* multiplied by assigned number of counting beads added based on lot *(C)* divided by sample volume *(D):*

$$\frac{A}{B}\times \frac{C}{D}=\text{concentration}\;\text{of}\;\text{sample}$$

### Statistical analysis

The Mann-Whitney *U* test was used to assess the difference between nonasthmatic controls and all patients as well as those with and without response at baseline. These data were expressed as means ± SDs. The differences between response status groups (response or nonresponse) throughout each time point (average of weeks −1 and 0 for baseline and weeks 6, 14, and 26) were determined by Wilcoxon matched-pairs signed rank test. Data were presented as Tukey-style box plots, with boxes binding interquartile range divided by medians.^43^ Multiple comparisons were adjusted for type I errors by applying the Bonferroni correction. To determine the clinical correlations of monocytes (Table I), a nonparametric Spearman correlation was calculated. All statistical analyses were performed by GraphPad Prism v10 software (GraphPad Software, La Jolla, Calif). Significance was set at *P* < .05.

**Table I:** Surface CD88 expression levels on blood monocytes associated with asthma symptoms

Characteristic	Spearman rho	*P* value
β-Agonist receipt per week vs CD88 MFI		
CD88 on CD14^+^	0.4374	.00378
CD88 on CD14^+^CD16^−^	0.4205	.00555
CD88 on CD14^+^CD16^+^	0.3003	.0476
CD88 on CD14^dim^CD16^+^	0.3889	.0091
ACT score vs CD88 MFI		
CD88 on CD14^+^	−0.2828	.0598
CD88 on CD14^+^CD16^−^	−0.2731	.0695
CD88 on CD14^+^CD16^+^	−0.2068	.1729
CD88 on CD14^dim^CD16^+^	−0.2499	.0978

MFI values were acquired after subtracting MFI values of control antibodies. Significance was set at *P* < .05. Spearman correlation test was performed without statistical correction.

## Results

### Omalizumab decreases mDC/pDC ratio in responsive disease

Because omalizumab treatment can decrease FcεRI expression on DCs^37^ and FcεRI expressed on DCs is known to deliver intracellular signals,^34^^,^^44^ we hypothesized that neutralizing serum IgE with omalizumab could alter the frequency and/or surface phenotypes of blood circulating DCs.

**Fig 1:**
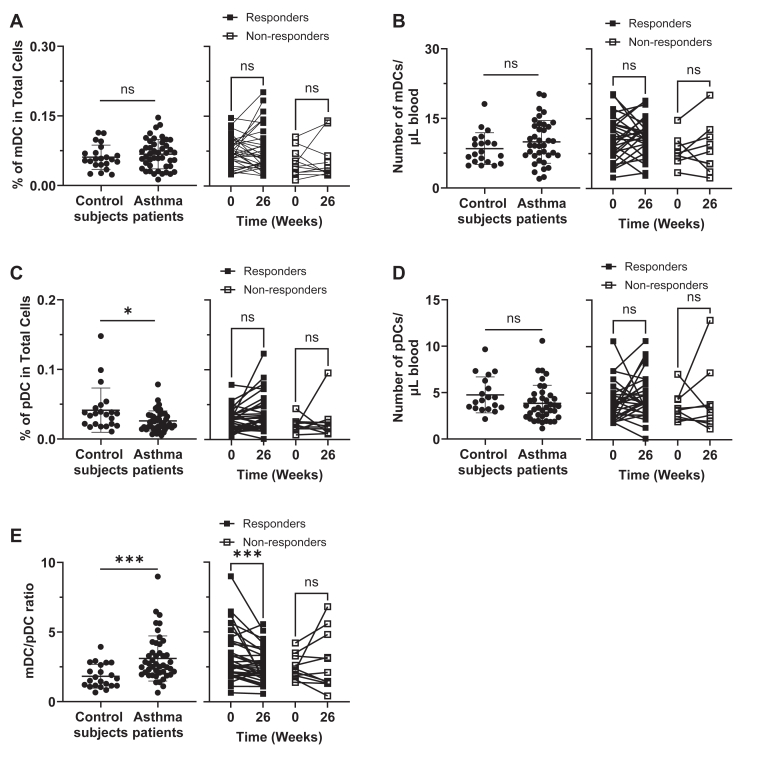
Moderate-to-severe asthma patients have aberrant mDC/pDC ratios that can be modified by anti-IgE treatment. Percentage **(A)** and number **(B)** of mDCs (Lin^−^HLA-DR^+^CD123^−^CD11c^+^) per microliter in whole blood from nonasthmatic control subjects and asthma patients *(left),* as well as those with response to omalizumab and those without response at baseline and week 26 *(right),* were assessed by flow cytometry. Percentage **(C)** and number **(D)** of pDCs (Lin^−^HLA-DR^+^CD123^+^CD11c^−^) per microliter of whole blood. Ratio of percentage of mDCs to pDCs **(E)**. Mann-Whitney *U* test *(left)* and Wilcoxon matched-pairs signed rank test *(right)* were used, respectively. Data for individual control subjects and asthma patients *(A-E, left)* are presented as means ± SDs. ∗*P* < .05, ∗∗*P* < .01, ∗∗∗*P* < .001, ∗∗∗∗*P* < .0001; *ns,* not significant.

We first compared the frequency of DC subsets (mDCs, CD141^+^ mDCs, and pDCs, gated as in Fig E1, *A*, in the Online Repository available at www.jaci-global.org) in the blood of nonasthmatic control subjects and moderate-to-severe asthma patients recruited in this study. Fig 1, *A* and *B, left,* show that nonasthmatic control subjects (n = 22) and moderate-to-severe asthma patients (n = 45) have similar percentages as well as actual numbers of mDCs per microliter of whole blood at baseline (week 0). The frequency of mDCs was not significantly altered by omalizumab treatment (Fig 1, *A* and *B, right*). A similar trend was observed in frequency and number of CD141^+^ mDCs (Fig E1, *B* and *C*).

In contrast to mDCs (Fig 1, *A* and *B*), asthma patients had lower frequency of pDCs than nonasthmatic control subjects (Fig 1, *C, left*). However, pDC counts per microliter of blood were not significantly different in the two groups (Fig 1, *D, left*). Although the percentages and numbers of pDC per microliter of blood were increased in some of the patients at week 26, such increases were not statistically significant (Fig 1, *C* and *D, right*).

Of interest, the mDC/pDC ratios acquired with the percentages of pDCs were greater in asthma patients than control subjects at baseline (Fig 1, *E, left*). In addition, omalizumab treatment decreased mDC/pDC ratio, especially in those with response to omalizumab. However, the mDC/pDCs ratio in those with response at week 26 (mean ± SD, 2.564 ± 1.190) was still higher than the mDC/pDC ratio in control subjects (1.823 ± 0.8530). Changes in the percentages and actual numbers of pDCs per microliter of blood from week 0 to week 26 are presented in Fig E2, *A* and *B,* in the Online Repository available at www.jaci-global.org. We noticed that the numbers of pDCs per microliter of blood increased by week 14 during omalizumab treatment (Fig E2, *B*). In line with the data in Fig 1, *E,* omalizumab decreased the mDC/pDC ratio from week 6, and this decrease was significant in those with response at week 26 (Fig E2, *C*).

Therefore, we concluded that moderate-to-severe adult asthma patients, compared to control subjects, have greater mDC/pDC ratios in their blood. In addition, such skewed mDC/pDC ratios in asthma patients could be redirected by omalizumab treatment.

### Omalizumab decreases surface FcεRI, CCR7, HLA-DR, and costimulatory molecule expression on mDCs

**Fig 2:**
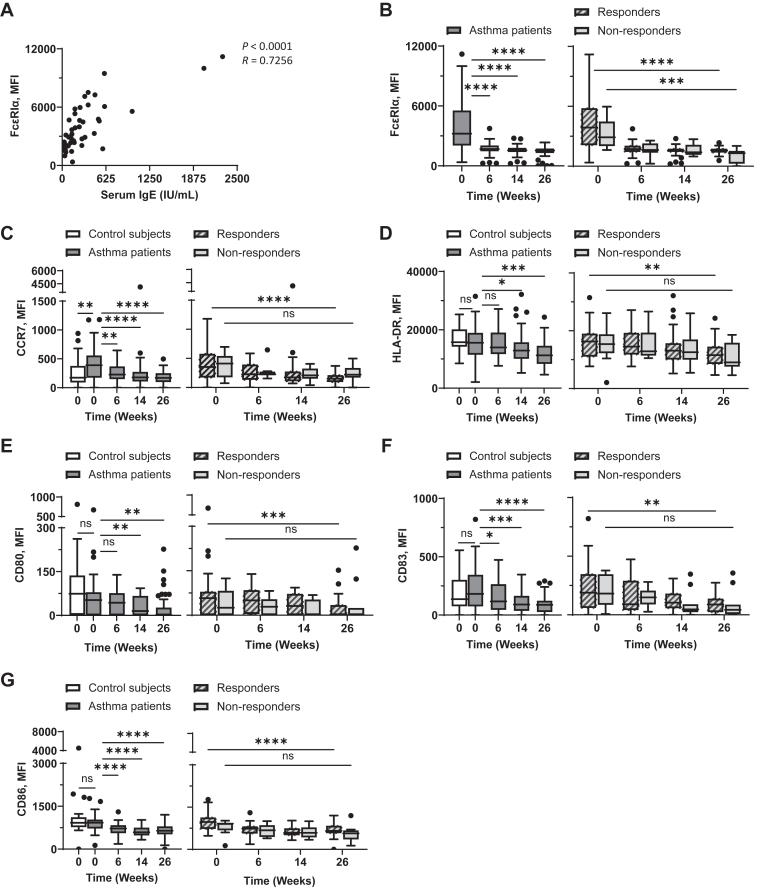
Omalizumab decreases surface expression levels of FcεRI, HLA-DR, CCR7, and costimulatory molecules on mDCs. Correlation between expression levels of FcεRIα on mDCs (Lin^−^HLA-DR^+^CD123^−^CD11c^+^) and serum IgE concentrations at week 0 was assessed **(A)**. mDCs in whole blood were stained, and surface expression level of FcεRI **(B)**, CCR7 **(C)**, HLA-DR **(D)**, CD80 **(E)**, CD83 **(F)**, and CD86 **(G)** were measured by flow cytometry at indicated time points. *Left,* Baseline expression levels of surface molecules on mDCs from nonasthmatic controls and asthma patients were compared, as well as during treatment for asthma patients. *Right,* Expression levels of surface molecules on mDCs of those with and those without response throughout treatment. Mann-Whitney *U* test was used to test difference between control subjects and asthma patients. Wilcoxon matched-pairs signed rank test was used to test difference between week 0 and week 26 of those with or without response. Data are expressed as Tukey-style box plots, with boxes binding interquartile range (IQR) divided by median. ∗*P* < .05, ∗∗*P* < .01, ∗∗∗*P* < .001, ∗∗∗∗*P* < .0001; *ns,* not significant.

We next tested whether omalizumab can alter surface phenotypes of blood mDCs. As shown in Fig 2, *A,* serum IgE levels correlated with surface expression levels of FcεRI on mDCs. In line with the data in previous studies,^37^^,^^45^ omalizumab decreased surface FcεRI levels on blood mDCs of asthma patients (Fig 2, *B, left*), although omalizumab could decrease FcεRI expression levels on mDCs of both those with and without response (Fig 2, *B, right*).

To test the effects of omalizumab treatment on additional surface phenotypes of mDCs, we measured the expression levels of CCR7, HLA-DR, CD80, CD83, and CD86 from week 0 (baseline) to week 26 (Fig 2, *C-G*). Similar to FcεRI, the expression levels of CCR7, HLA-DR, and the costimulatory molecules we tested (CD80, CD83, and CD86) were decreased throughout the omalizumab treatment period (Fig 2, *C-G*). It was also of note that mDCs in patients expressed higher levels of CCR7 than those in nonasthmatic control subjects (Fig 2, *C, left*). Fig E3, in the Online Repository available at www.jaci-global.org, presents surface expression levels of FcεRI, CCR7, HLA-DR, CD80, CD83, and CD86 on mDCs in individual patients (separately for those with and without response) measured at baseline and at week 26.

In conclusion, although omalizumab treatment does not alter the frequency or number of blood mDCs (Fig 1, *A* and *B*), it significantly reduces the surface expression levels of FcεRI, HLA-DR, CCR7, and costimulatory molecules on blood mDCs. Because DCs are the major immune inducers, these data (Fig 2) suggest that such omalizumab-induced downregulation of those molecules could affect the clinical outcomes of omalizumab treatment in asthma patients.

### Omalizumab-mediated downregulation of surface FcεRI, CCR7, HLA-DR, and costimulatory molecule expression on pDCs

**Fig 3:**
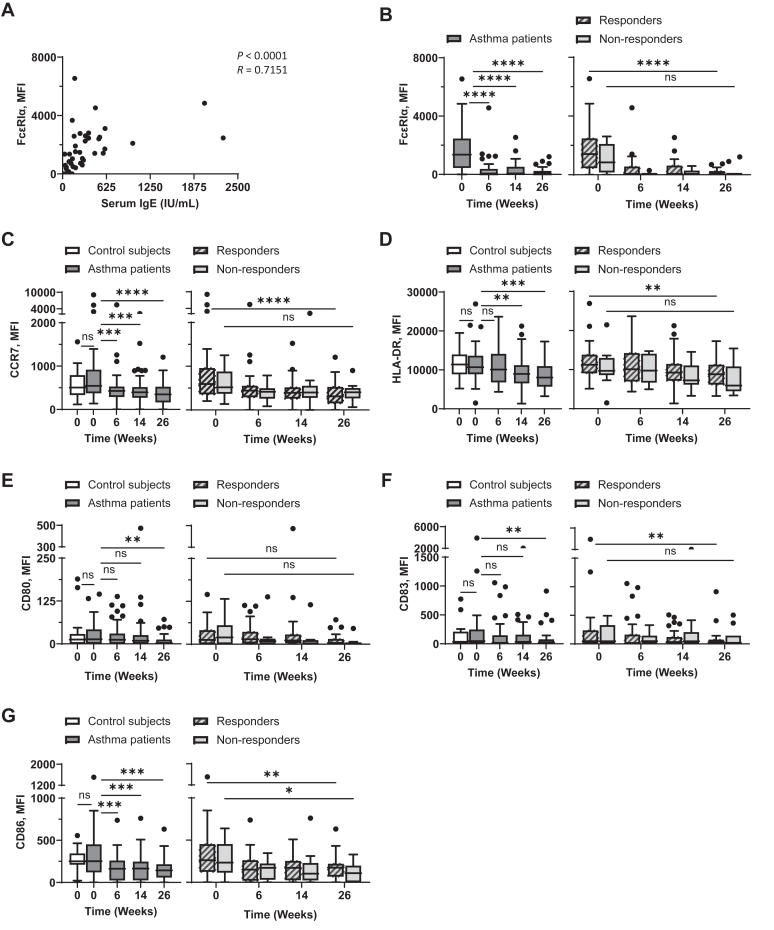
Omalizumab decreases surface expression levels of FcεRI, HLA-DR, CCR7, and costimulatory molecules on pDCs. Correlation between expression levels of FcεRIα on pDCs (Lin^−^HLA-DR^+^CD123^+^CD11c^−^) and serum IgE concentrations at week 0 was assessed **(A)**. pDCs in whole blood were stained, and surface expression levels of FcεRI **(B)**, CCR7 **(C)**, HLA-DR **(D)**, CD80 **(E)**, CD83 **(F)**, and CD86 **(G)** were measured by flow cytometry at indicated time points. *Left,* Baseline expression levels of surface molecules on pDCs of nonasthmatic controls and asthma patients were compared, as well as during treatment for asthma patients. *Right,* Expression levels of surface molecules on pDCs of those with and without response throughout treatment. Mann-Whitney *U* test was used to test difference between control subjects and asthma patients. Wilcoxon matched-pairs signed rank test was used to test differences between week 0 and week 26 for those with and without response. Data are expressed as Tukey-style box plots, with boxes binding interquartile range (IQR) divided by median. ∗*P* < .05, ∗∗*P* < .01, ∗∗∗*P* < .001, ∗∗∗∗*P* < .0001; *ns,* not significant.

In line with previously published data,^46^ pDCs in asthma patients expressed surface Fc epsilon receptor Iα (FcεRIα), and the expression levels of FcεRIα correlated with serum IgE levels (Fig 3, *A*). Omalizumab resulted in a significant reduction of FcεRI expression on pDCs in asthma patients from baseline to week 26 (Fig 3, *B, left*). This decrease in FcεRIα was significant in those with response to omalizumab from baseline to week 26 (Fig 3, *B, right*).

Consistent with the decrease in FcεRI, the expressions of CCR7, HLA-DR, and costimulatory molecules (CD80, CD83, CD86) were also downregulated throughout the treatment period, with this decline being significant from baseline to week 26 (Fig 3, *C-G, left*). Fig 3, *C-G, right,* further shows that such decreases of CCR7, HLA-DR, CD83, and CD86 were significant in the response group from baseline to week 26. Fig E4, in the Online Repository available at www.jaci-global.org, presents expression levels of FcεRI, CCR7, HLA-DR, and costimulatory molecules on pDCs from individual asthma patients measured at weeks 0 and 26.

Taking these data together (Fig 1, Fig 2, Fig 3), we concluded that omalizumab treatment results in decreased FcεRI expression as well as decreased expression of CCR7, HLA-DR, and costimulatory molecules on both blood mDC and pDCs of asthma patients. Such decreases from baseline to week 26 were significant in those with omalizumab-responsive disease.

### Asthma patients exhibit increased frequency of CD14^+^ monocytes unaffected by omalizumab treatment

**Fig 4:**
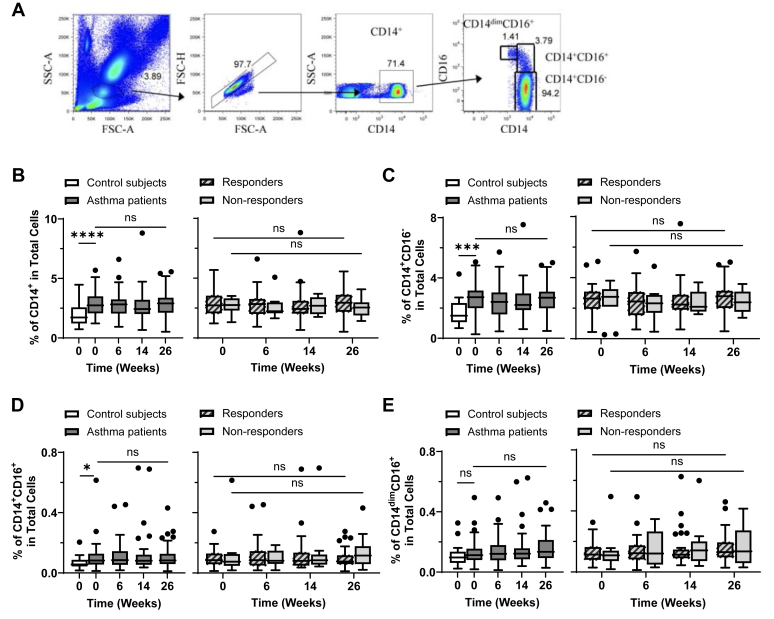
Asthma patients have increased frequency of blood-circulating CD14^+^ monocytes. Gating strategy **(A)** for total CD14^+^, CD14^+^CD16^+^, CD14^+^CD16^−^, and CD14^dim^CD16^+^ monocytes from stained whole blood. Baseline (week 0) frequency of total CD14^+^**(B)**, CD14^+^CD16^−^**(C)**, CD14^+^CD16^+^**(D)**, and CD14^dim^CD16^+^**(E)** monocytes in blood of nonasthmatic control subjects and asthma patients were compared (*B-E, left*). Frequency of individual monocyte subsets was measured at indicated time points throughout omalizumab treatment for all asthma patients (*B-E, left*) and those with response versus those with no response (*B-E, right*). Mann-Whitney *U* test was used to test difference between control subjects and asthma patients. Wilcoxon matched-pairs signed rank test was used to test differences between week 0 and week 26 of those with or without response. Data are expressed as Tukey-style box plots, with boxes binding interquartile range (IQR) divided by median. ∗*P* < .05, ∗∗*P* < .01, ∗∗∗*P* < .001, ∗∗∗∗*P* < .0001; *ns,* not significant.

Monocytes can also contribute to allergic responses not only by presenting allergens to T cells but also by producing various proinflammatory cytokines and chemokines. A previous study also reported that omalizumab decreases surface FcεRI expression level on monocytes.^47^ However, dysregulation of different subsets of monocytes, along with the effects of omalizumab on the frequency and phenotype of individual monocyte subsets in asthma patients, have not been previously investigated. Therefore, we investigated whether the percentages and numbers as well as surface phenotypes of total CD14^+^, CD14^+^CD16^−^ (classical monocyte), CD14^+^CD16^+^ (intermediate monocyte), and CD14^dim^CD16^+^ (nonclassical monocyte) subsets (Fig 4, *A*), were affected by omalizumab treatment over time.

We found that at baseline, asthma patients exhibited higher percentages of total CD14^+^ as well as CD14^+^CD16^−^ and CD14^+^CD16^+^ monocyte subsets compared to nonasthmatic control subjects (Fig 4, *B-E, left*). Similarly, asthma patients also had increased numbers of total CD14^+^, CD14^+^CD16^−^, and CD14^+^CD16^+^ monocytes per microliter of blood compared to nonasthmatic control subjects (see Fig E5, *A-C, left,* in the Online Repository available at www.jaci-global.org). No difference was observed between those with and without response in terms of the frequencies or numbers of monocyte subsets per microliter of blood from week 0 to week 26 (Fig 4, *B-E, right,* and Fig E5*, right*). Asthma patients exhibited increased numbers of CD14^+^CD2^hi^ monocytes (Fig E5, *E, left*), but omalizumab treatment did not significantly alter their numbers in both those with and without response (Fig E5, *E, right*).

Therefore, we concluded that moderate-to-severe asthma patients, compared to nonasthmatic control subjects, have increased percentages and numbers of total CD14^+^, CD14^+^CD16^−^ (classical monocytes), CD14^+^CD16^+^ (intermediate monocytes), and CD14^+^CD2^hi^ blood circulating monocytes. Omalizumab did not alter the frequency of any of the monocyte subsets tested in this study.

### Omalizumab alters surface phenotypes of blood monocytes

We next measured the expression levels of FcεRI, CCR7, HLA-DR, and the complement receptor C5a (CD88) on individual monocyte subsets at baseline and during omalizumab treatment. CD88 was included as one of the surface phenotypes of interest because it is a chemotactic receptor that can promote monocyte migration toward inflammatory sites.^48^

#### CD14^+^CD16^−^ classical monocytes

**Fig 5:**
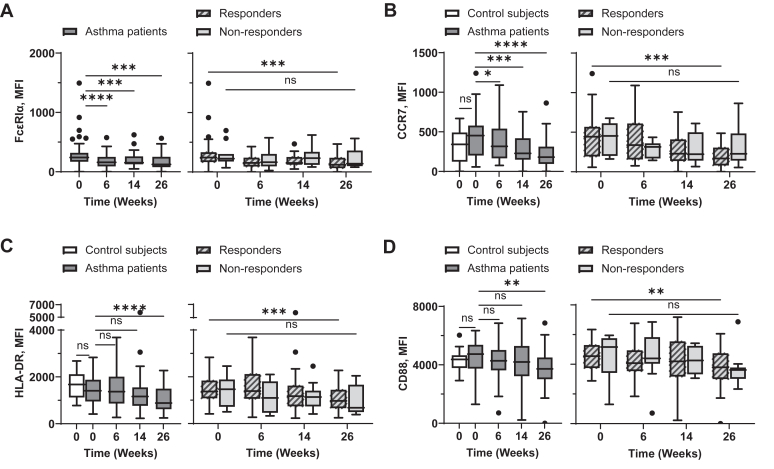
Omalizumab decreases surface expression levels of FcεRI as well as CCR7, HLA-DR, and CD88 on CD14^+^CD16^−^ blood monocytes in asthma patients, especially those with response to omalizumab. Surface expression levels of FcεRIα **(A)**, CCR7 **(B)**, HLA-DR **(C)**, and CD88 **(D)** on CD14^+^CD16^−^ blood monocytes were measured with flow cytometry at indicated time points. *Left,* Data of nonasthmatic controls compared to asthma patients at baseline (week −1/0) as well as expression levels of individual surface molecules on CD14^+^CD16^−^ blood monocytes from all asthma patients at indicated time points. *Right,* Data of those with and without omalizumab response. Mann-Whitney *U* test was used to test difference between control subjects and asthma patients at baseline. Wilcoxon matched-pairs signed rank test was used to calculate significance between week 0 and week 26 of those with or without response. Data are expressed as Tukey-style box plots, with boxes binding interquartile range (IQR) divided by median. ∗*P* < .05, ∗∗*P* < .01, ∗∗∗*P* < .001, ∗∗∗∗*P* < .0001; *ns,* not significant.

Although the surface expression levels of FcεRI on CD14^+^CD16^−^ monocytes (mean fluorescence intensity [MFI] 301.1 ± 37.42, Fig 5, *A*) were lower than those on mDCs (MFI 3986 ± 368.8, Fig 2, *B*), omalizumab could still decrease FcεRI expression levels on CD14^+^CD16^−^ monocytes (Fig 5, *A, left*). The decrease of FcεRI expression level from baseline to week 26 was significant in those with response to omalizumab (Fig 5, *A, right*). We also found that omalizumab decreased expression levels of CCR7, HLA-DR, and CD88 on CD14^+^CD16^−^ monocytes (Fig 5, *B-D, left*). Although such decreases from baseline to week 26 were more statistically significant in those with response than in those without response (Fig 5, *B-D, right*), the average MFI values of individual molecules were decreased in both those with and without response. Expression levels of FcεRI, CCR7, HLA-DR, and CD88 on CD14^+^CD16^−^ monocytes from individual asthma patients measured at weeks 0 and 26 are presented in Fig E6 in the Online Repository available at www.jaci-global.org.

#### CD14^+^CD16^+^ intermediate monocytes

**Fig 6:**
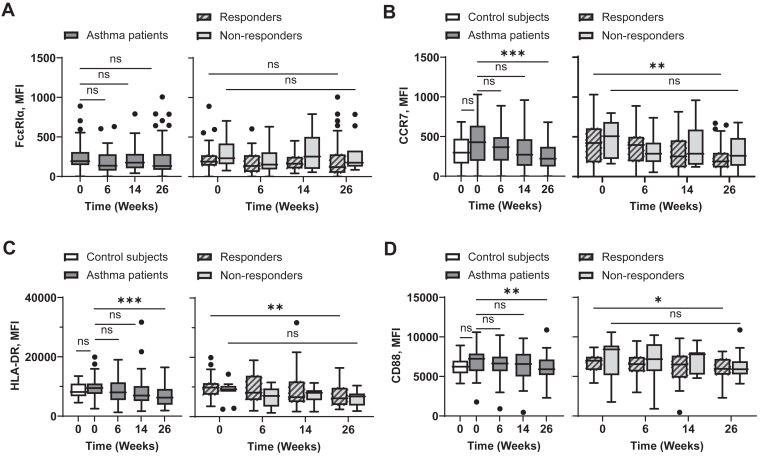
Omalizumab decreases surface expression levels of FcεRI as well as CCR7, HLA-DR, and CD88 on CD14^+^CD16^+^ blood monocyte in asthma patients, especially those with response to omalizumab. Surface expression levels of FcεRI **(A)**, CCR7 **(B)**, HLA-DR **(C)**, and CD88 **(D)** on CD14^+^CD16^+^ blood monocytes were measured with flow cytometry at indicated time points. *Left,* Data of nonasthmatic controls compared to asthma patients at baseline (week −1/0) as well as expression levels of individual surface molecules on CD14^+^CD16^+^ blood monocyte from all asthma patients at indicated time points. *Right,* Data of those with response to omalizumab and those with no response. Mann-Whitney *U* test was used to test difference between control subjects and asthma patients at baseline. Wilcoxon matched-pairs signed rank test was used to calculate significance between week 0 and week 26 of those with or without response. Data are expressed as Tukey-style box plots, with boxes binding interquartile range (IQR) divided by median. ∗*P* < .05, ∗∗*P* < .01, ∗∗∗*P* < .001, ∗∗∗∗*P* < .0001; *ns,* not significant.

CD14^+^CD16^+^ monocytes (Fig 6, *A, left*) in asthma patients expressed similar baseline levels of FcεRI as that observed on CD14^+^CD16^−^ monocytes (Fig 5, *A, left*) at baseline. In addition, there was no difference between those with response to omalizumab and those without response at baseline (Fig 6, *A, right*). Although the average MFI values of FcεRI decreased over time after omalizumab treatment in those with and without response, these changes were not statistically significant (Fig 6, *A*). However, omalizumab treatment significantly decreased CCR7, HLA-DR, and CD88 expression levels by week 26 (Fig 6, *B-D, left*). Such decreases from baseline to week 26 were significant in those with response (Fig 6, *B-D, right*). It was also noted that CD14^+^CD16^+^ monocytes expressed increased levels of both HLA-DR and CD88, but not FcεRI or CCR7, compared to CD14^+^CD16^−^ classical monocytes. The average MFI values of CCR7, HLA-DR, and CD88 were slightly higher in asthma patients than in nonasthmatic control subjects, but there was no significant difference between the two groups. Expression levels of FcεRI, CCR7, HLA-DR, and CD88 on CD14^+^CD16^+^ monocytes from individual those with response and those without response measured at weeks 0 and 26 are presented in Fig E7 in the Online Repository available at www.jaci-global.org.

#### CD14^dim^CD16^+^ nonclassical monocytes

**Fig 7:**
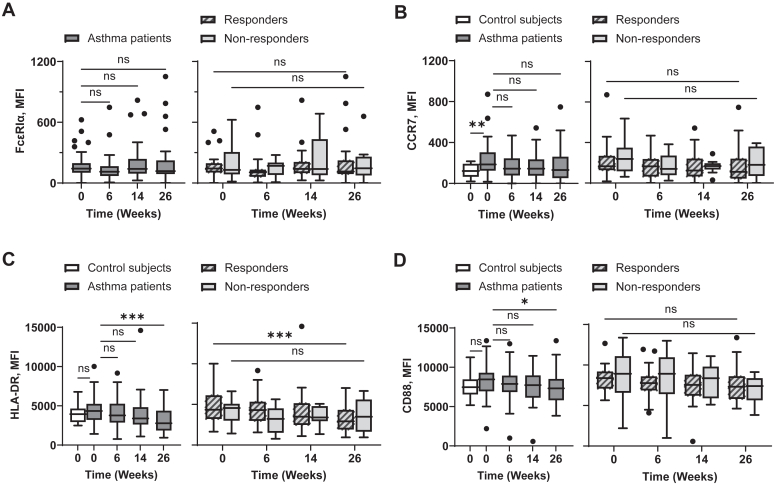
Omalizumab decreases surface expression levels of HLA-DR and CD88 on CD14^dim^CD16^+^ blood monocyte in asthma patients. Surface expression levels of FcεRI **(A)**, CCR7 **(B)**, HLA-DR **(C)**, and CD88 **(D)** on CD14^dim^CD16^+^ blood monocytes were measured with flow cytometry at indicated time points. *Left,* Data of nonasthmatic controls compared to asthma patients at baseline (week −1/0) as well as expression levels of individual surface molecules on CD14^dim^CD16^+^ blood monocyte from all asthma patients at indicated time points. *Right,* Data of those with response to omalizumab and those with nonresponse. Mann-Whitney *U* test was used to test difference between control subjects and asthma patients at baseline. Wilcoxon matched-pairs signed rank test was used to calculate significance between week 0 and week 26 of those with response or no response. Data are expressed as Tukey-style box plots, with boxes binding interquartile range (IQR) divided by median. ∗*P* < .05, ∗∗*P* < .01, ∗∗∗*P* < .001, ∗∗∗∗*P* < .0001; *ns,* not significant.

Compared to CD14^+^CD16^−^ classical monocytes (MFI 301.1 ± 37.42), CD14^dim^CD16^+^ nonclassical monocytes had lower FcεRI expression (MFI 169.5 ± 18.50) at week 0 (Fig 7, *A, left*). Omalizumab treatment did not significantly alter FcεRI expression levels in those with or without response (Fig 7, *A, right*). Although CD14^dim^CD16^+^ monocytes in asthma patients expressed higher levels of CCR7 than control subjects (Fig 7, *B, left*), there were no significant effects of omalizumab treatment over time in those with or without response (Fig 7, *B, right*). However, omalizumab decreased HLA-DR expression (Fig 7, *C, left*) on CD14^dim^CD16^+^ monocytes, which was only significant in the response group (Fig 7, *C, right*). Compared to CD14^+^CD16^−^ classical monocytes (MFI 4554 ± 160.2; Fig 5, *D, left*), CD14^dim^CD16^+^ nonclassical monocytes expressed increased levels of CD88 (MFI 8336 ± 300.4; Fig 7, *D, left*). Omalizumab treatment resulted in the decrease of CD88 expression levels at week 26 when tested with all asthma patients (Fig 7, *D, left*). Although CD88 expression levels were decreased in both those with and without response, significant changes were not observed in either group (Fig 7, *D, right*). Expression levels of FcεRI, CCR7, HLA-DR, and CD88 on CD14^dim^CD16^+^ nonclassical monocytes from those with and without response are presented in Fig E8 in the Online Repository available at www.jaci-global.org.

Taken together, our findings indicate that omalizumab treatment leads to decreased activation of blood monocytes, as evidenced by reduced expression of CCR7, HLA-DR, and CD88 across all 3 monocyte subsets from baseline to week 26 of treatment.

### CD88 expression on blood monocytes are clinically correlated with asthma symptoms

To further examine the clinical relevance of the decrease in CD88 expression on monocyte subsets, we assessed the correlations between CD88 expression levels on monocyte subsets and asthma symptoms by performing a nonparametric Spearman correlation test. The relationship between CD88 expression and two clinical variables, β-agonist receipt per week and ACT score, is presented in Table I.

A significant positive correlation was observed between CD88 expression on total CD14^+^ monocytes and β-agonist receipt per week. This positive correlation was also significant across the 3 subtypes of CD14^+^ monocytes: CD14^+^CD16^−^, CD14^+^CD16^+^, and CD14^dim^CD16^+^.

Although a negative correlation between the expression levels of CD88 and ACT scores was observed across the 3 subtypes of CD14^+^ monocytes, none of these correlations reached statistical significance.

## Discussion

This study reports new insights into mechanisms of action of omalizumab that are clinically relevant. The therapeutic effects of omalizumab have been mainly explained by the reduction of surface FcεR1 expression on basophils and mast cells^15^, ^16^, ^17^, ^18^, ^19^ as well as APCs,^9^^,^^10^ resulting in decreased secretion of inflammatory mediators, in addition to reduced allergen uptake and presentation to T cells, respectively. We found that omalizumab therapy significantly downregulates several key surface molecules, including CCR7, HLA-DR, CD80, and CD86 on both mDCs and pDCs, which are the major APCs. Omalizumab also decreases mDC/pDC ratios in the blood of those with response to omalizumab. In addition to DCs, surface expression of CCR7, HLA-DR, and CD88 on monocytes was also significantly decreased by omalizumab treatment.

Whole blood staining revealed that the frequency and actual numbers of mDC per microliter of blood in asthma patients and control subjects were similar at baseline. The percentages and actual numbers of mDC in the whole blood staining were not affected by omalizumab therapy. In contrast to mDCs, asthma patients had slightly decreased percentages of pDCs compared to control subjects (Fig 1, *C*). Matsuda et al^49^ reported in 2003 that asthma patients had an increased frequency of pDCs in their peripheral blood compared to healthy control subjects. All patients in that study had well-controlled asthma and were nonsmokers, whereas the moderate-to-severe asthma patients recruited onto this study were prescribed inhaled corticosteroids, which can substantially decrease pDC frequency.^50^^,^^51^

With the decreased frequency of pDCs in asthma patients, the mDC/pDC ratio was significantly elevated at baseline compared to nonasthmatic controls (Fig 1, *E*). This altered mDC/pDC ratio was also previously reported in chronic obstructive pulmonary disease patients.^52^ However, it was of note that omalizumab treatment reduced the mDC/pDC ratio over time in those with response; this is likely due to a selective increase of the frequency of pDCs (Fig E2, *A*). Although additional studies are warranted to further understand this omalizumab-induced increase of circulating blood pDC frequency, potential roles of pDCs^28^ in the suppression of allergic inflammatory response need to be studied further.

Alterations of surface phenotypes of DCs by omalizumab treatment have not been previously reported. Although additional studies are needed, the reduction of CCR7, HLA-DR, and costimulatory molecules (CD80, CD83, CD86), particularly in those with omalizumab-responsive disease, likely reflects decreased activation in DCs, which is thought to contribute to the anti-inflammatory effects of omalizumab. Such a decrease in CCR7, HLA-DR, and costimulatory molecules on DCs and monocytes are thought to be the results of the decrease of serum IgE–mediated activation signals delivered via FcεRI.^53^ Our findings align with this mechanism: we observed that FcεRI expression on mDCs and pDCs was positively correlated with serum IgE concentration (Fig 2, *A,* and Fig 3, *A*) and that omalizumab decreased surface FcεRI expression. This decrease in FcεRI likely contributes to reduced allergen presentation and T-cell activation, supporting the immunomodulatory role of omalizumab in asthma. CCR7 is crucial for DC migration to lymph nodes, and its downregulation suggests that omalizumab treatment may reduce the capacity of DCs to migrate and initiate T-cell responses.^36^ Similarly, the downregulation of HLA-DR and costimulatory molecules reflects diminished allergen-specific T-cell responses. Collectively, data from this study suggest that omalizumab-induced downregulation of CCR7, HLA-DR, and costimulatory molecules on DCs, which are the major APCs, could dampen the allergic inflammatory response in patients. Beyond its action on IgE neutralization followed by reduced signaling via FcεRI,^15^, ^16^, ^17^, ^18^, ^19^ which might contribute to the altered phenotypes of DCs and monocytes, omalizumab also resulted in the restoration of immunologic imbalance in severe allergic asthma patients.^54^ In our recent study, we observed that omalizumab treatment could decrease serum cytokine (including granulocyte-macrophage colony-stimulating factor, IL-5, and IL-7) and chemokine (including chemokine C-C motif ligand [CCL] 2, CCL4, and CCL8) levels (unpublished data). However, additional studies are warranted to investigate potential alternative mechanisms for the omalizumab-induced alterations of DC and monocyte phenotypes.

Monocytes, which contribute to allergic lung inflammation, also exhibited altered activation in response to omalizumab. Surface expression of CCR7, HLA-DR, and CD88 was downregulated over the course of omalizumab treatment, similar to changes seen in DCs. Again, these alterations were more pronounced in those with omalizumab-responsive disease, suggesting that omalizumab modulates monocyte activation, potentially reducing monocytes’ ability to promote allergic inflammation. The reduction in CD88 expression suggests that omalizumab treatment may impair, at least in part, the ability of monocytes to migrate to sites of inflammation, as CD88 contribute to monocyte chemotaxis.^55^^,^^56^ This is in line with the observed downregulation of CCR7, which regulates monocyte migration to lymph nodes. Therefore, these findings suggest that omalizumab might reduce allergic inflammatory response by limiting the trafficking and activation of peripheral APCs.

The clinical relevance of these findings is further supported by our observation that CD88 expression on monocytes correlates with asthma symptoms, including β-agonist receipt (Table I). Specifically, increased CD88 expression on CD14^+^ monocytes was associated with more frequent β-agonist receipt. The negative correlation between CD88 expression and ACT scores indicates that monocyte activation may also influence overall asthma exacerbation (Table I) because higher CD88 levels were associated with lower ACT scores. Therefore, our data indicate that CD88 expression could serve as a useful biomarker for monitoring asthma activity and predicting omalizumab’s treatment outcomes. However, additional studies with larger numbers of patients are warranted to determine its clinical utility. For example, the CD88 expression levels might also vary among patients because of their biological variabilities or other factors.

One of the limitations of this study is the relatively small sample size of patients with no response to omalizumab, which may have limited the statistical power to detect significant changes in some markers. Future studies with larger cohorts are needed to confirm these findings and to explore additional biomarkers that could predict response to omalizumab more accurately. Furthermore, although our study provides valuable insights into the peripheral blood immune profiles of asthma patients, it remains to be seen whether similar alterations occur in the lower airways, where the primary inflammatory processes in asthma take place.

Our study shows associations between omalizumab treatment and changes in DC and monocyte phenotypes, but establishing firm causality is challenging because of the potential confounding factors. Concurrent medications like corticosteroids can induce immune cell apoptosis and suppress antigen presentation.^57^ Additionally, factors like disease severity, age, and comorbidities could further influence immune modulation. Aging is associated with immunosenescence, including decreased antigen-presenting capacity, altered cytokine production, and reduced FcεRI expression on DCs.^58^ Future studies should carefully document and control for these variables to better isolate omalizumab’s effects.

In conclusion, our findings suggest that omalizumab (anti-IgE) treatment reduces the activation of DCs and monocytes in asthma patients by downregulating not only surface FcεRI but also several key surface receptors, including CCR7, HLA-DR, and costimulatory molecules that contribute to allergic inflammatory responses elicited by DCs and monocytes. Overall, these effects were significant in those with response to omalizumab, indicating that it may exert its therapeutic effects by modulating APC function, thereby limiting allergen-specific T-cell responses.

**Key messages**

• Omalizumab decreases mDC/pDC ratio in the blood of moderate-to-severe asthma patients, especially in those with disease responsive to omalizumab.
• Omalizumab decreases surface expression levels of not only FcεRI but also HLA-DR, CCR7, and costimulatory molecules on DC and monocyte subsets.
• Omalizumab-induced downregulation of CD88 expression on monocytes is associated with asthma symptoms.
• Data from this study highlight new insights into the mechanisms of action of omalizumab in asthma patients.

## Disclosure statement

Supported by the 10.13039/100008925American Asthma Foundation (AAF15-0038, S.O.), Investigator-Initiated Study from 10.13039/100004328Genentech (ML28019, S.O.), and the 10.13039/100000871Mayo Clinic.

Disclosure of potential conflict of interest: The authors declare that they have no relevant conflicts of interest.
