# Supplementary material for: Omalizumab controls surface phenotypes of dendritic cells and monocytes in asthma
Source: J Allergy Clin Immunol Glob. 2025 Jun 23;4(3):100523. doi: 10.1016/j.jacig.2025.100523 (PMC12281943; doi:10.1016/j.jacig.2025.100523)
Supplement: Supplementary Figs 1 to 8 [file mmc1.pptx]

## Slide 1
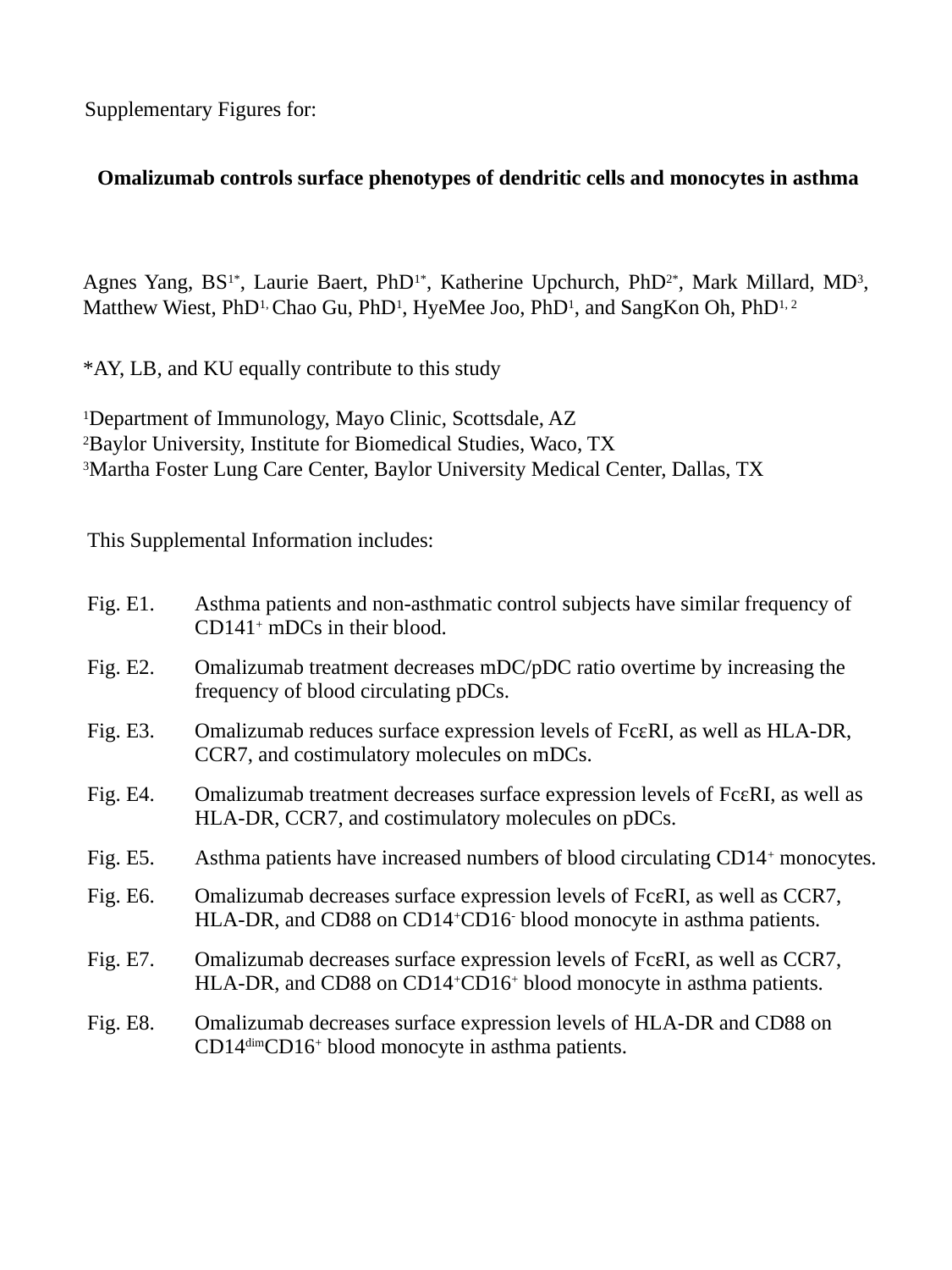

Supplementary Figures for:
Omalizumab controls surface phenotypes of dendritic cells and monocytes in asthma
Agnes Yang, BS1*, Laurie Baert, PhD1*, Katherine Upchurch, PhD2*, Mark Millard, MD3, Matthew Wiest, PhD1, Chao Gu, PhD1, HyeMee Joo, PhD1, and SangKon Oh, PhD1, 2
*AY, LB, and KU equally contribute to this study
1Department of Immunology, Mayo Clinic, Scottsdale, AZ
2Baylor University, Institute for Biomedical Studies, Waco, TX
3Martha Foster Lung Care Center, Baylor University Medical Center, Dallas, TX
| This Supplemental Information includes: | |
| --- | --- |
| Fig. E1. | Asthma patients and non-asthmatic control subjects have similar frequency of CD141+ mDCs in their blood. |
| Fig. E2. | Omalizumab treatment decreases mDC/pDC ratio overtime by increasing the frequency of blood circulating pDCs. |
| Fig. E3. | Omalizumab reduces surface expression levels of FcɛRI, as well as HLA-DR, CCR7, and costimulatory molecules on mDCs. |
| Fig. E4. | Omalizumab treatment decreases surface expression levels of FcɛRI, as well as HLA-DR, CCR7, and costimulatory molecules on pDCs. |
| Fig. E5. | Asthma patients have increased numbers of blood circulating CD14+ monocytes. |
| Fig. E6. | Omalizumab decreases surface expression levels of FcɛRI, as well as CCR7, HLA-DR, and CD88 on CD14+CD16- blood monocyte in asthma patients. |
| Fig. E7. | Omalizumab decreases surface expression levels of FcɛRI, as well as CCR7, HLA-DR, and CD88 on CD14+CD16+ blood monocyte in asthma patients. |
| Fig. E8. | Omalizumab decreases surface expression levels of HLA-DR and CD88 on CD14dimCD16+ blood monocyte in asthma patients. |
| | |
| | |

## Slide 2
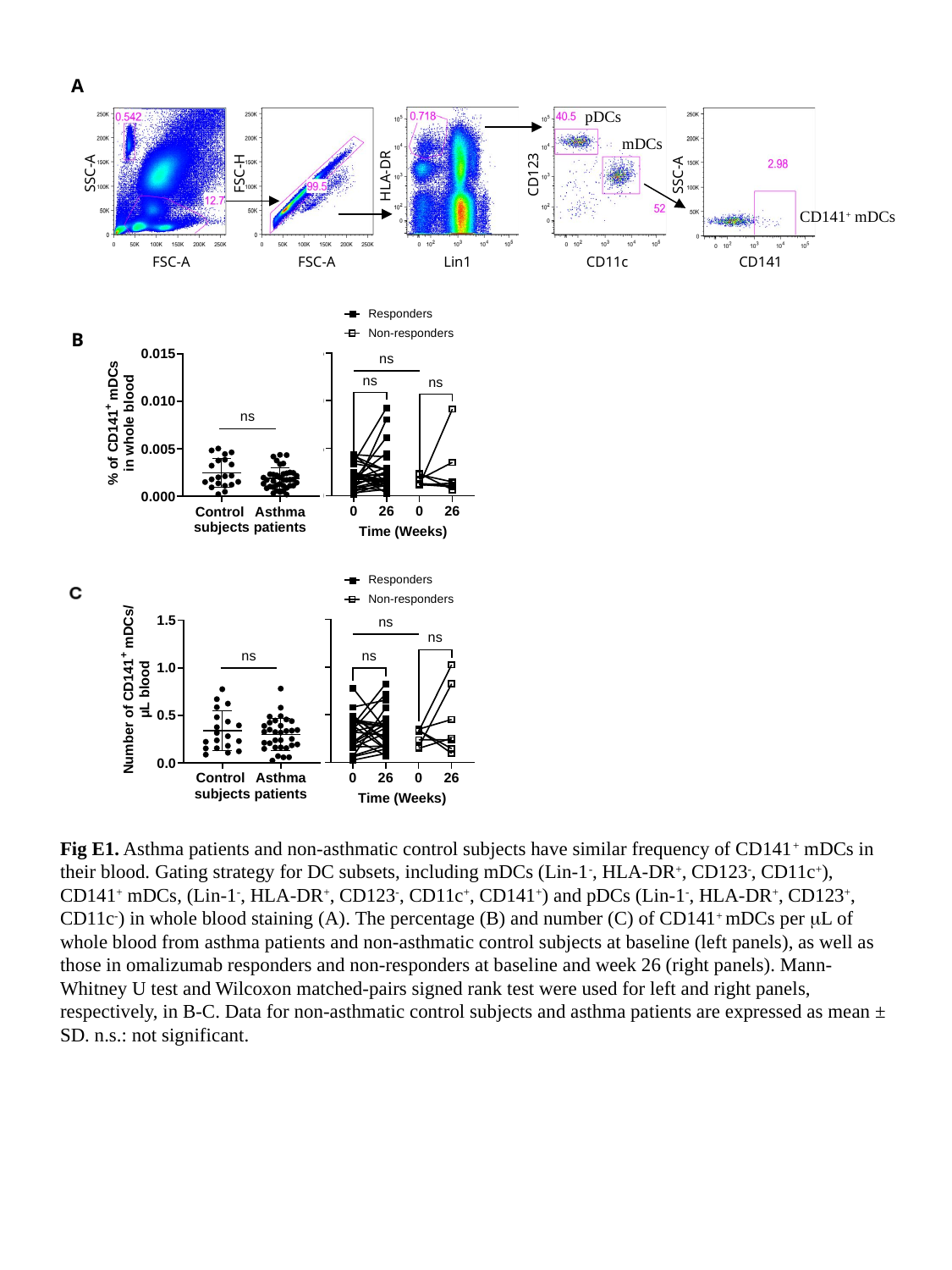

A
pDCs
FSC-H
SSC-A
CD123
SSC-A
HLA-DR
FSC-A
Lin1
CD11c
FSC-A
CD141
mDCs
CD141+ mDCs
B
Fig E1. Asthma patients and non-asthmatic control subjects have similar frequency of CD141+ mDCs in their blood. Gating strategy for DC subsets, including mDCs (Lin-1-, HLA-DR+, CD123-, CD11c+), CD141+ mDCs, (Lin-1-, HLA-DR+, CD123-, CD11c+, CD141+) and pDCs (Lin-1-, HLA-DR+, CD123+, CD11c-) in whole blood staining (A). The percentage (B) and number (C) of CD141+ mDCs per μL of whole blood from asthma patients and non-asthmatic control subjects at baseline (left panels), as well as those in omalizumab responders and non-responders at baseline and week 26 (right panels). Mann-Whitney U test and Wilcoxon matched-pairs signed rank test were used for left and right panels, respectively, in B-C. Data for non-asthmatic control subjects and asthma patients are expressed as mean ± SD. n.s.: not significant.

## Slide 3
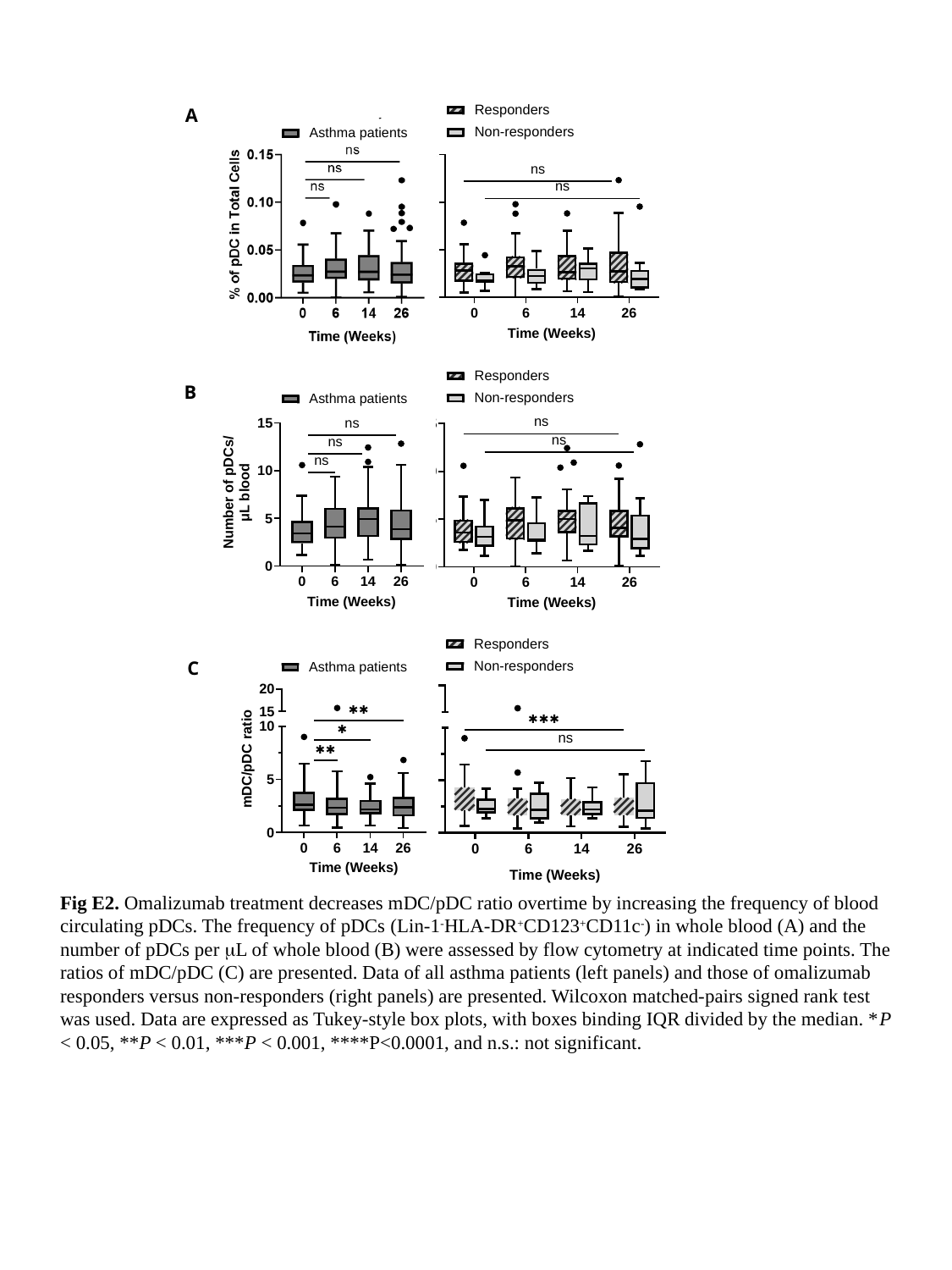

A
B
C
Fig E2. Omalizumab treatment decreases mDC/pDC ratio overtime by increasing the frequency of blood circulating pDCs. The frequency of pDCs (Lin-1-HLA-DR+CD123+CD11c-) in whole blood (A) and the number of pDCs per μL of whole blood (B) were assessed by flow cytometry at indicated time points. The ratios of mDC/pDC (C) are presented. Data of all asthma patients (left panels) and those of omalizumab responders versus non-responders (right panels) are presented. Wilcoxon matched-pairs signed rank test was used. Data are expressed as Tukey-style box plots, with boxes binding IQR divided by the median. *P < 0.05, **P < 0.01, ***P < 0.001, ****P<0.0001, and n.s.: not significant.

## Slide 4
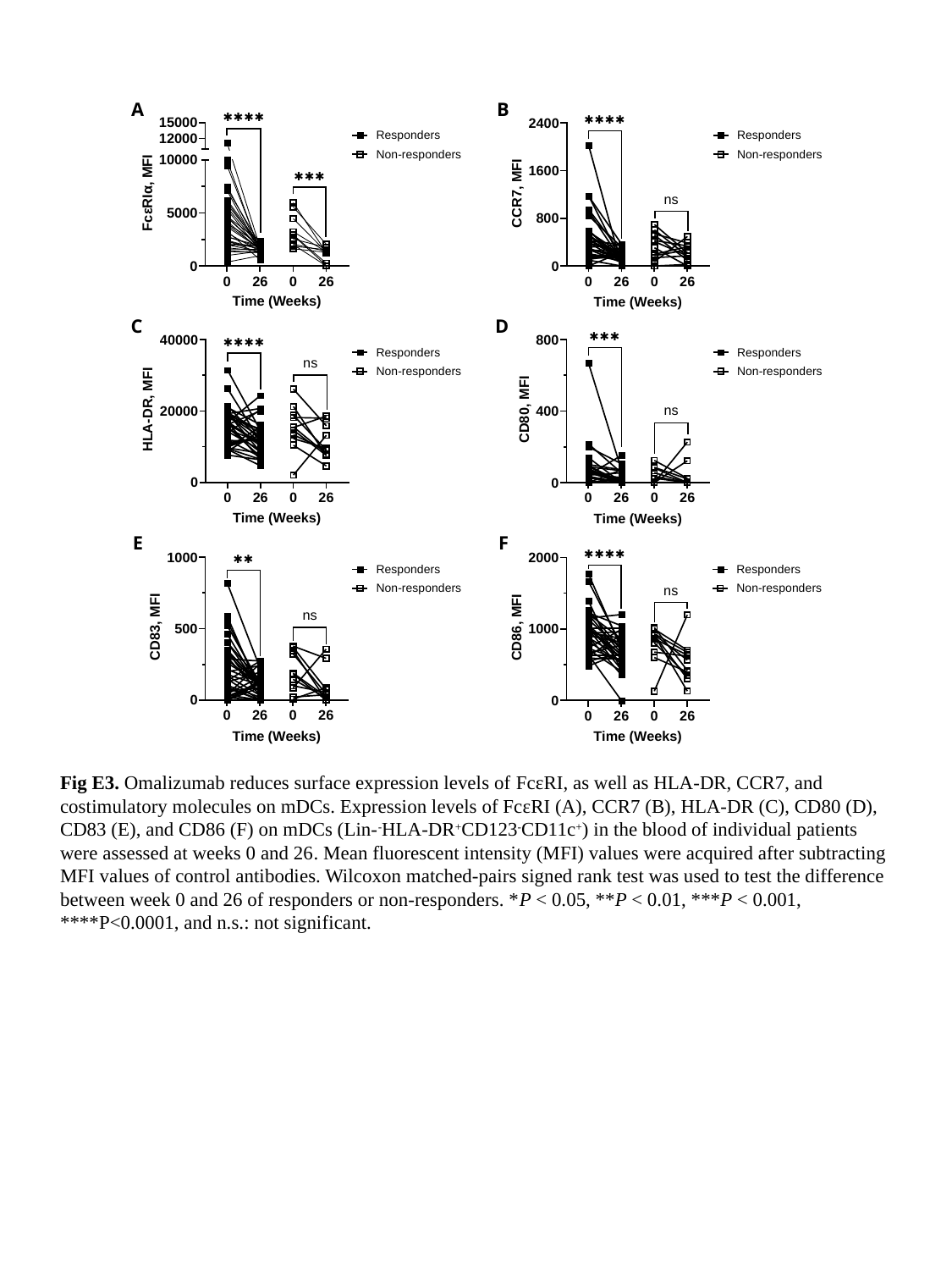

A
B
C
D
E
F
Fig E3. Omalizumab reduces surface expression levels of FcɛRI, as well as HLA-DR, CCR7, and costimulatory molecules on mDCs. Expression levels of FcɛRI (A), CCR7 (B), HLA-DR (C), CD80 (D), CD83 (E), and CD86 (F) on mDCs (Lin--HLA-DR+CD123-CD11c+) in the blood of individual patients were assessed at weeks 0 and 26. Mean fluorescent intensity (MFI) values were acquired after subtracting MFI values of control antibodies. Wilcoxon matched-pairs signed rank test was used to test the difference between week 0 and 26 of responders or non-responders. *P < 0.05, **P < 0.01, ***P < 0.001, ****P<0.0001, and n.s.: not significant.

## Slide 5
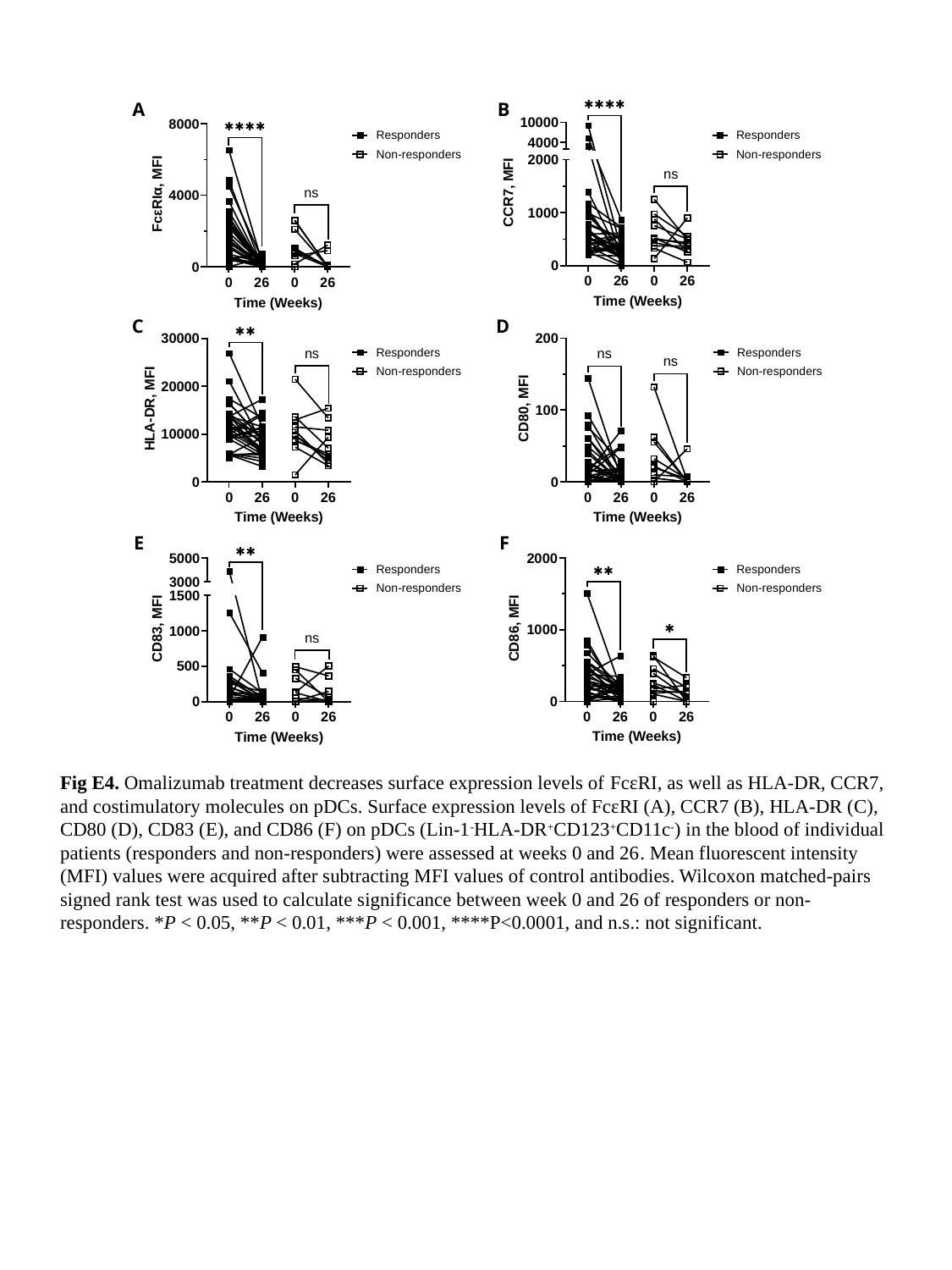

A
B
C
D
E
F
Fig E4. Omalizumab treatment decreases surface expression levels of FcɛRI, as well as HLA-DR, CCR7, and costimulatory molecules on pDCs. Surface expression levels of FcɛRI (A), CCR7 (B), HLA-DR (C), CD80 (D), CD83 (E), and CD86 (F) on pDCs (Lin-1-HLA-DR+CD123+CD11c-) in the blood of individual patients (responders and non-responders) were assessed at weeks 0 and 26. Mean fluorescent intensity (MFI) values were acquired after subtracting MFI values of control antibodies. Wilcoxon matched-pairs signed rank test was used to calculate significance between week 0 and 26 of responders or non-responders. *P < 0.05, **P < 0.01, ***P < 0.001, ****P<0.0001, and n.s.: not significant.

## Slide 6
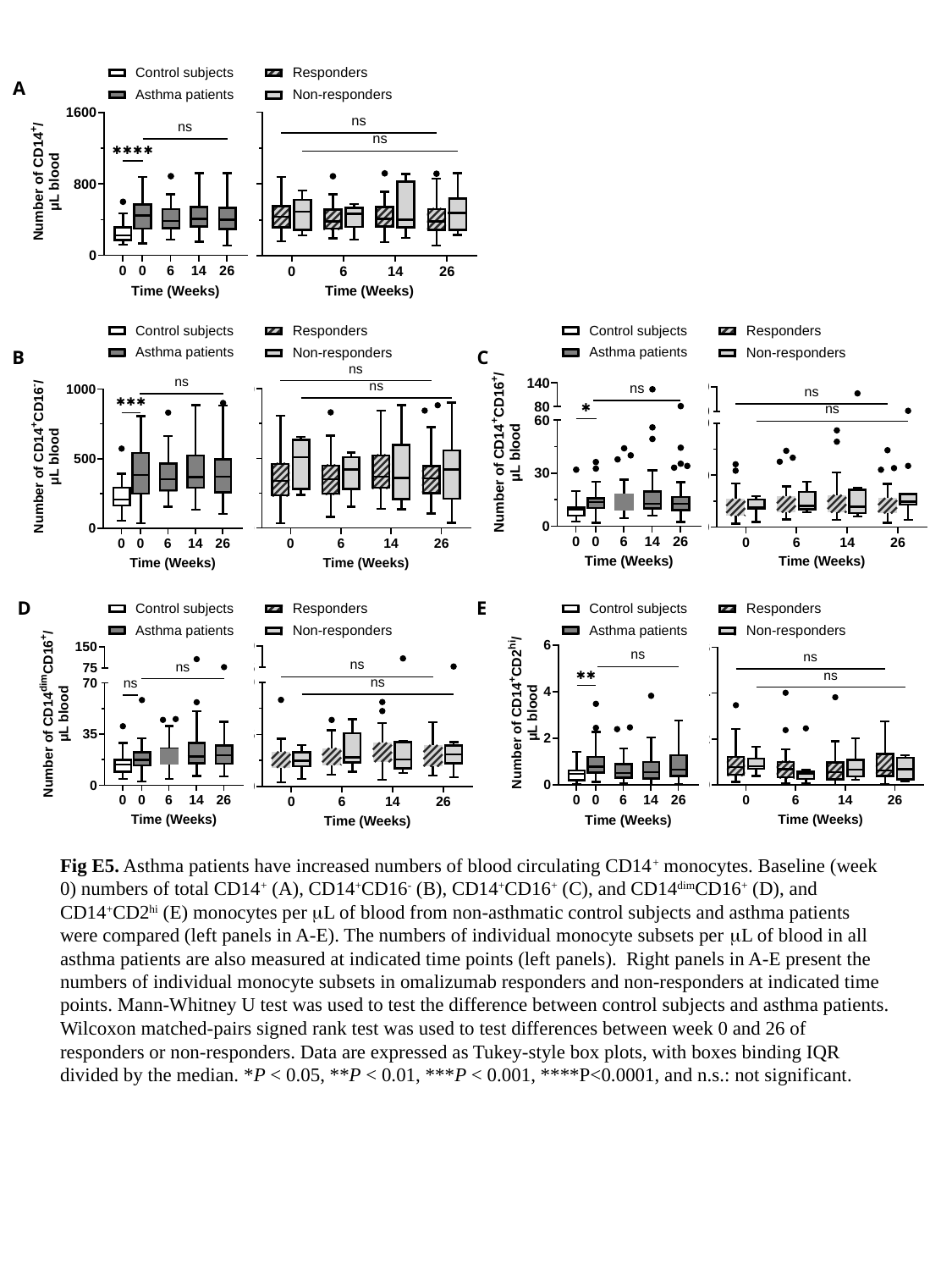

A
B
C
D
E
Fig E5. Asthma patients have increased numbers of blood circulating CD14+ monocytes. Baseline (week 0) numbers of total CD14+ (A), CD14+CD16- (B), CD14+CD16+ (C), and CD14dimCD16+ (D), and CD14+CD2hi (E) monocytes per mL of blood from non-asthmatic control subjects and asthma patients were compared (left panels in A-E). The numbers of individual monocyte subsets per mL of blood in all asthma patients are also measured at indicated time points (left panels). Right panels in A-E present the numbers of individual monocyte subsets in omalizumab responders and non-responders at indicated time points. Mann-Whitney U test was used to test the difference between control subjects and asthma patients. Wilcoxon matched-pairs signed rank test was used to test differences between week 0 and 26 of responders or non-responders. Data are expressed as Tukey-style box plots, with boxes binding IQR divided by the median. *P < 0.05, **P < 0.01, ***P < 0.001, ****P<0.0001, and n.s.: not significant.

## Slide 7
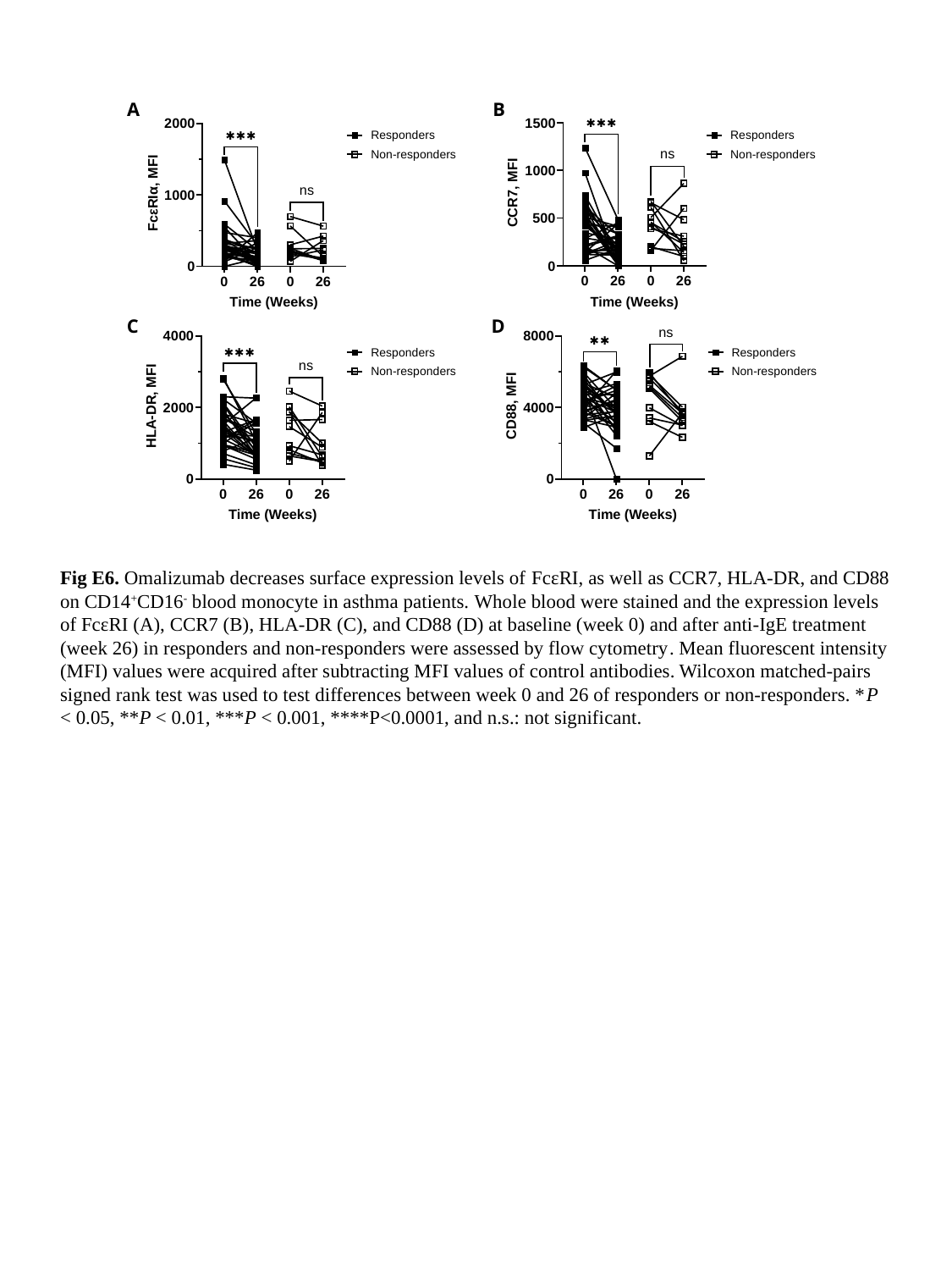

A
B
C
D
Fig E6. Omalizumab decreases surface expression levels of FcɛRI, as well as CCR7, HLA-DR, and CD88 on CD14+CD16- blood monocyte in asthma patients. Whole blood were stained and the expression levels of FcɛRI (A), CCR7 (B), HLA-DR (C), and CD88 (D) at baseline (week 0) and after anti-IgE treatment (week 26) in responders and non-responders were assessed by flow cytometry. Mean fluorescent intensity (MFI) values were acquired after subtracting MFI values of control antibodies. Wilcoxon matched-pairs signed rank test was used to test differences between week 0 and 26 of responders or non-responders. *P < 0.05, **P < 0.01, ***P < 0.001, ****P<0.0001, and n.s.: not significant.

## Slide 8
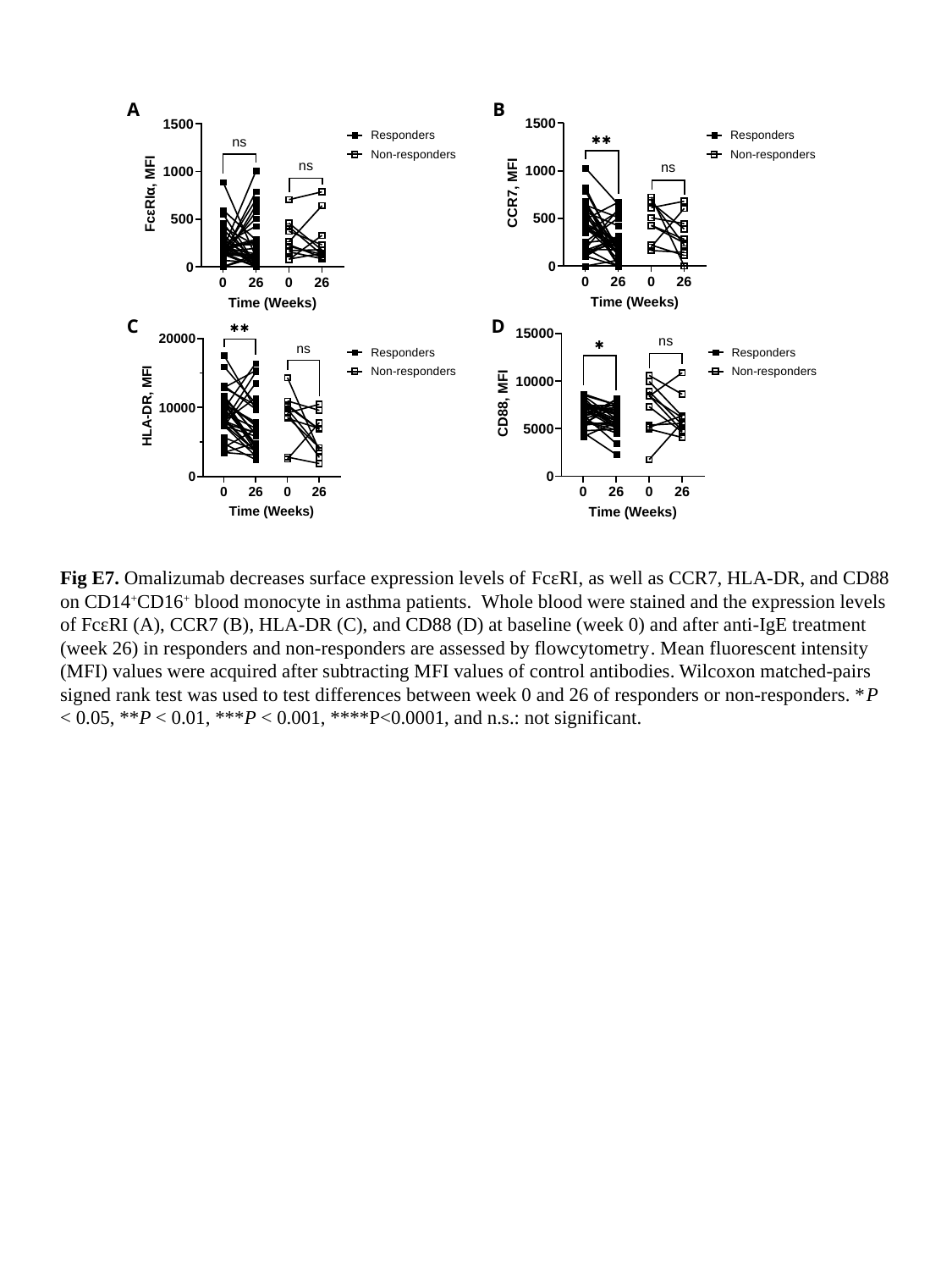

A
B
C
D
Fig E7. Omalizumab decreases surface expression levels of FcɛRI, as well as CCR7, HLA-DR, and CD88 on CD14+CD16+ blood monocyte in asthma patients. Whole blood were stained and the expression levels of FcɛRI (A), CCR7 (B), HLA-DR (C), and CD88 (D) at baseline (week 0) and after anti-IgE treatment (week 26) in responders and non-responders are assessed by flowcytometry. Mean fluorescent intensity (MFI) values were acquired after subtracting MFI values of control antibodies. Wilcoxon matched-pairs signed rank test was used to test differences between week 0 and 26 of responders or non-responders. *P < 0.05, **P < 0.01, ***P < 0.001, ****P<0.0001, and n.s.: not significant.

## Slide 9
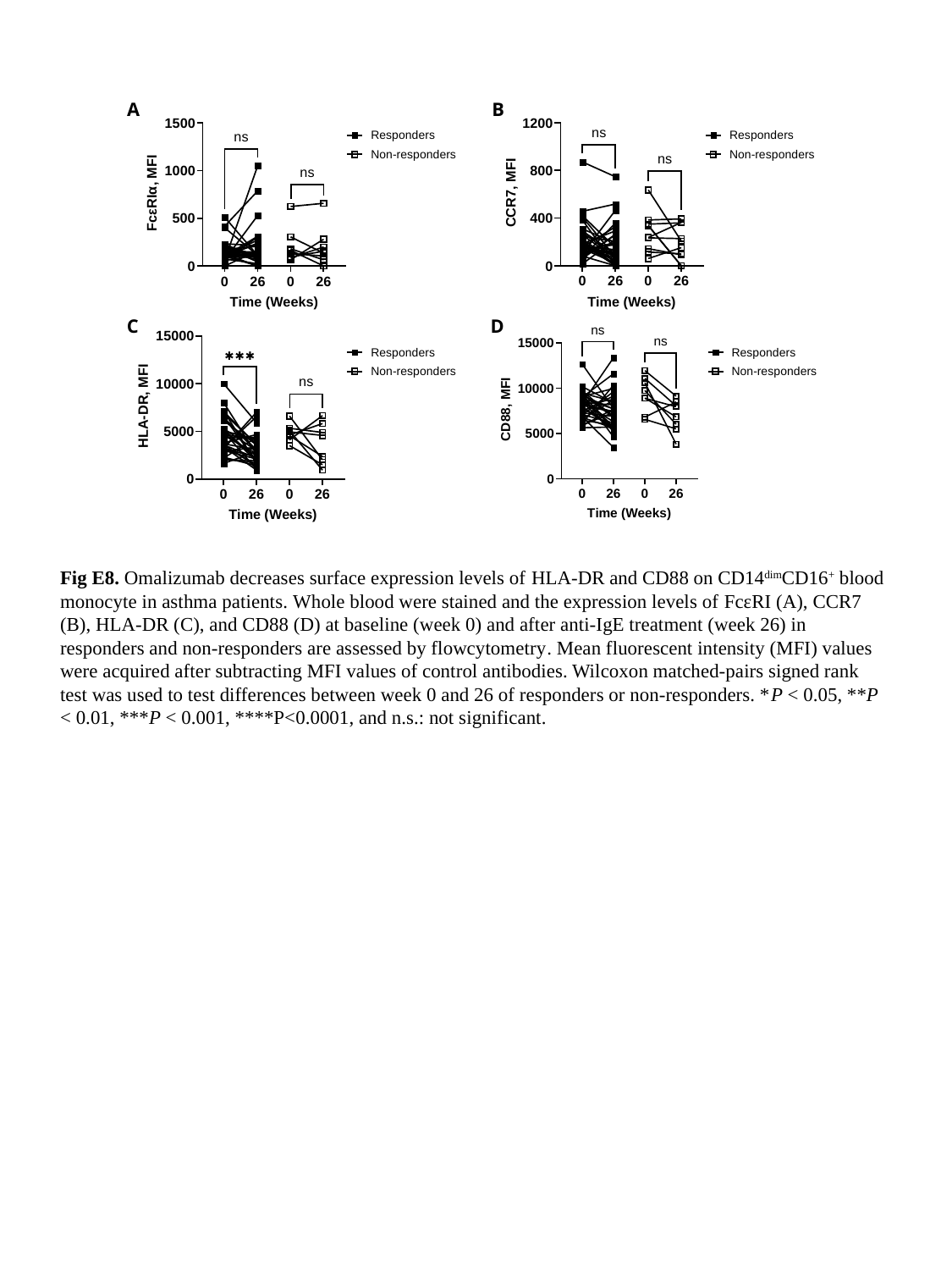

A
B
C
D
Fig E8. Omalizumab decreases surface expression levels of HLA-DR and CD88 on CD14dimCD16+ blood monocyte in asthma patients. Whole blood were stained and the expression levels of FcɛRI (A), CCR7 (B), HLA-DR (C), and CD88 (D) at baseline (week 0) and after anti-IgE treatment (week 26) in responders and non-responders are assessed by flowcytometry. Mean fluorescent intensity (MFI) values were acquired after subtracting MFI values of control antibodies. Wilcoxon matched-pairs signed rank test was used to test differences between week 0 and 26 of responders or non-responders. *P < 0.05, **P < 0.01, ***P < 0.001, ****P<0.0001, and n.s.: not significant.
