# Supplementary material for: Omalizumab controls surface phenotypes of dendritic cells and monocytes in asthma
Source: J Allergy Clin Immunol Glob. 2025 Jun 23;4(3):100523. doi: 10.1016/j.jacig.2025.100523 (PMC12281943; doi:10.1016/j.jacig.2025.100523)

## Slide 1
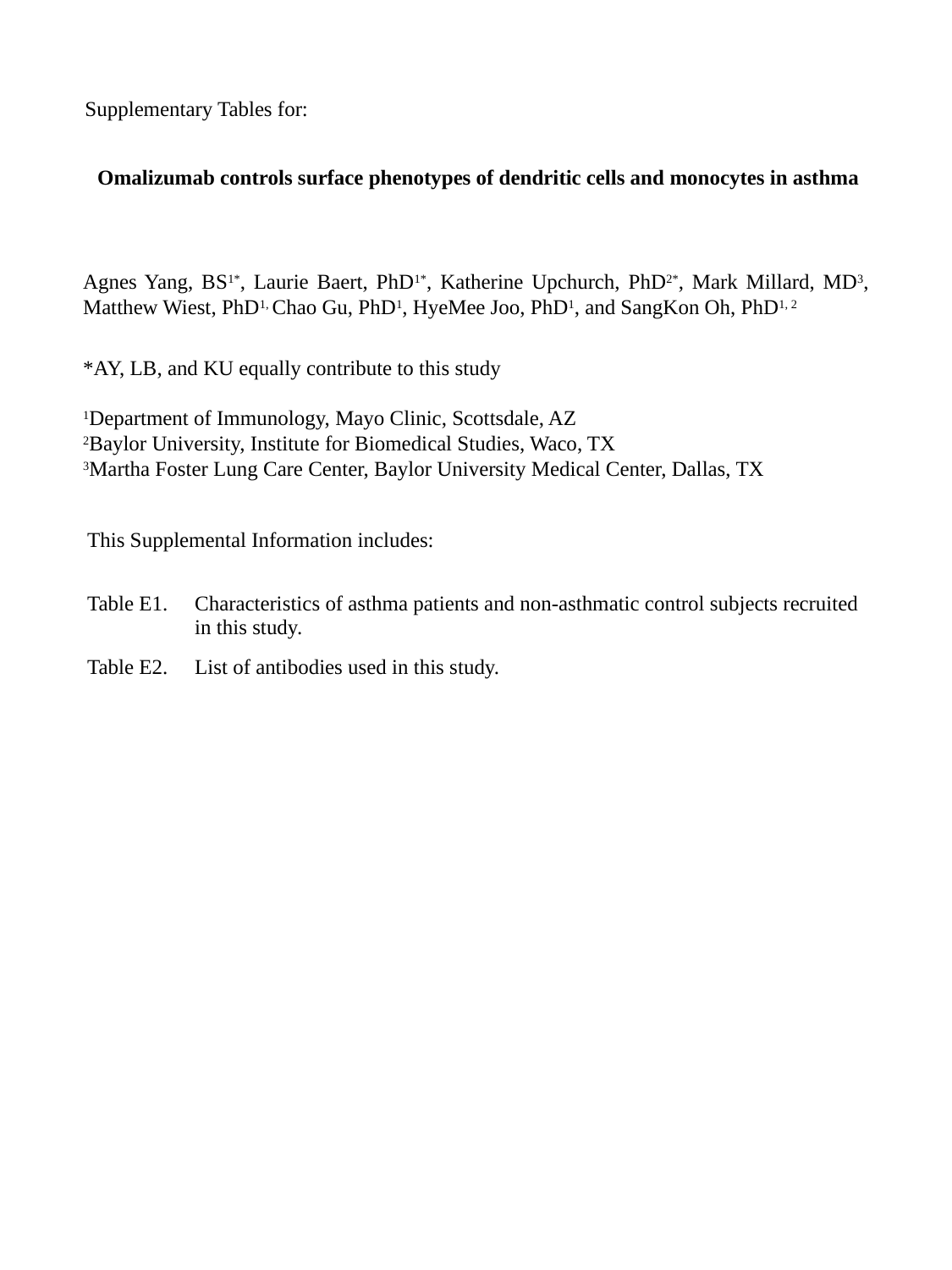

Supplementary Tables for:
Omalizumab controls surface phenotypes of dendritic cells and monocytes in asthma
Agnes Yang, BS1*, Laurie Baert, PhD1*, Katherine Upchurch, PhD2*, Mark Millard, MD3, Matthew Wiest, PhD1, Chao Gu, PhD1, HyeMee Joo, PhD1, and SangKon Oh, PhD1, 2
*AY, LB, and KU equally contribute to this study
1Department of Immunology, Mayo Clinic, Scottsdale, AZ
2Baylor University, Institute for Biomedical Studies, Waco, TX
3Martha Foster Lung Care Center, Baylor University Medical Center, Dallas, TX
| This Supplemental Information includes: | |
| --- | --- |
| Table E1. | Characteristics of asthma patients and non-asthmatic control subjects recruited in this study. |
| Table E2. | List of antibodies used in this study. |

## Slide 2
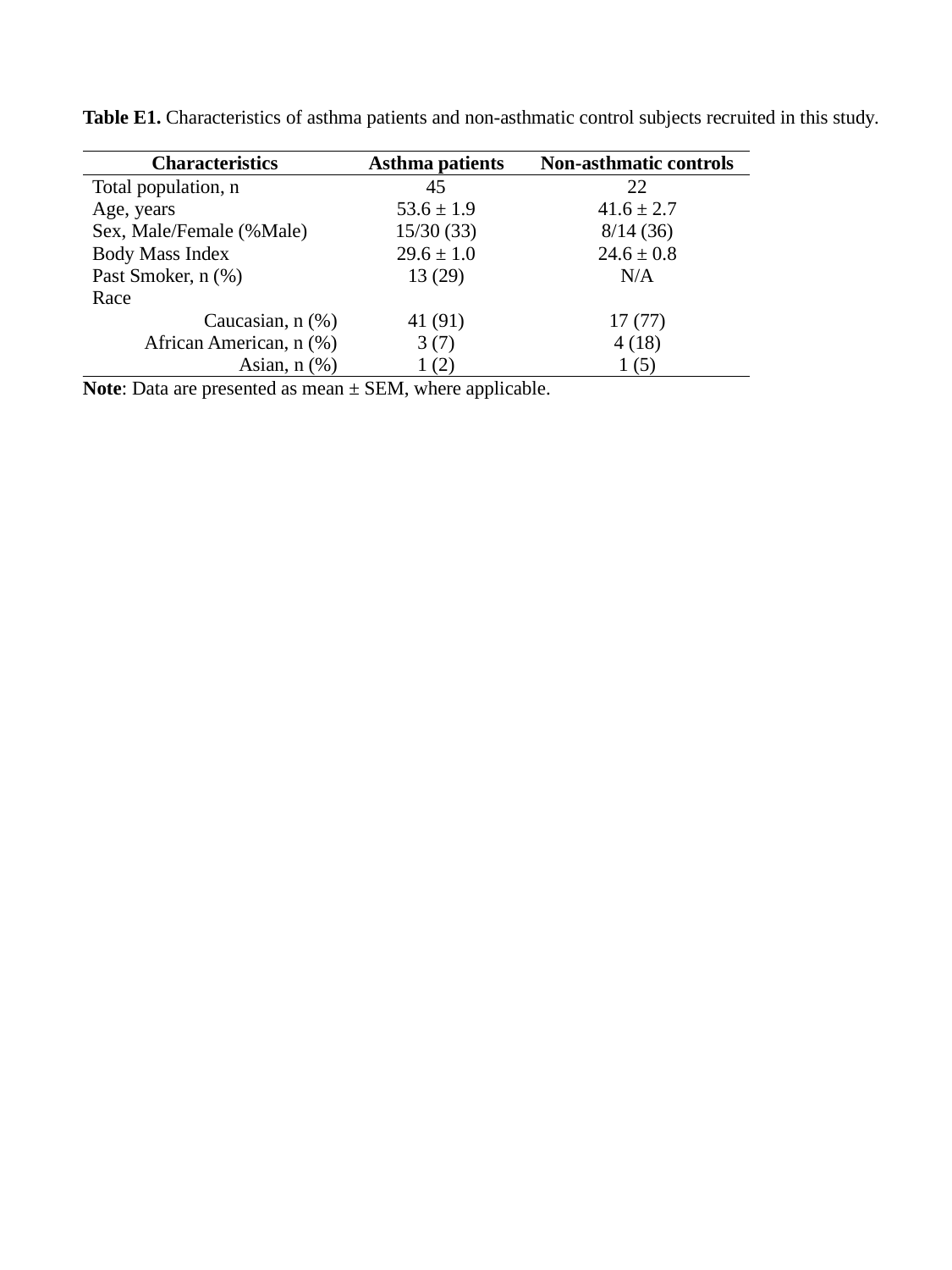

## Slide 3
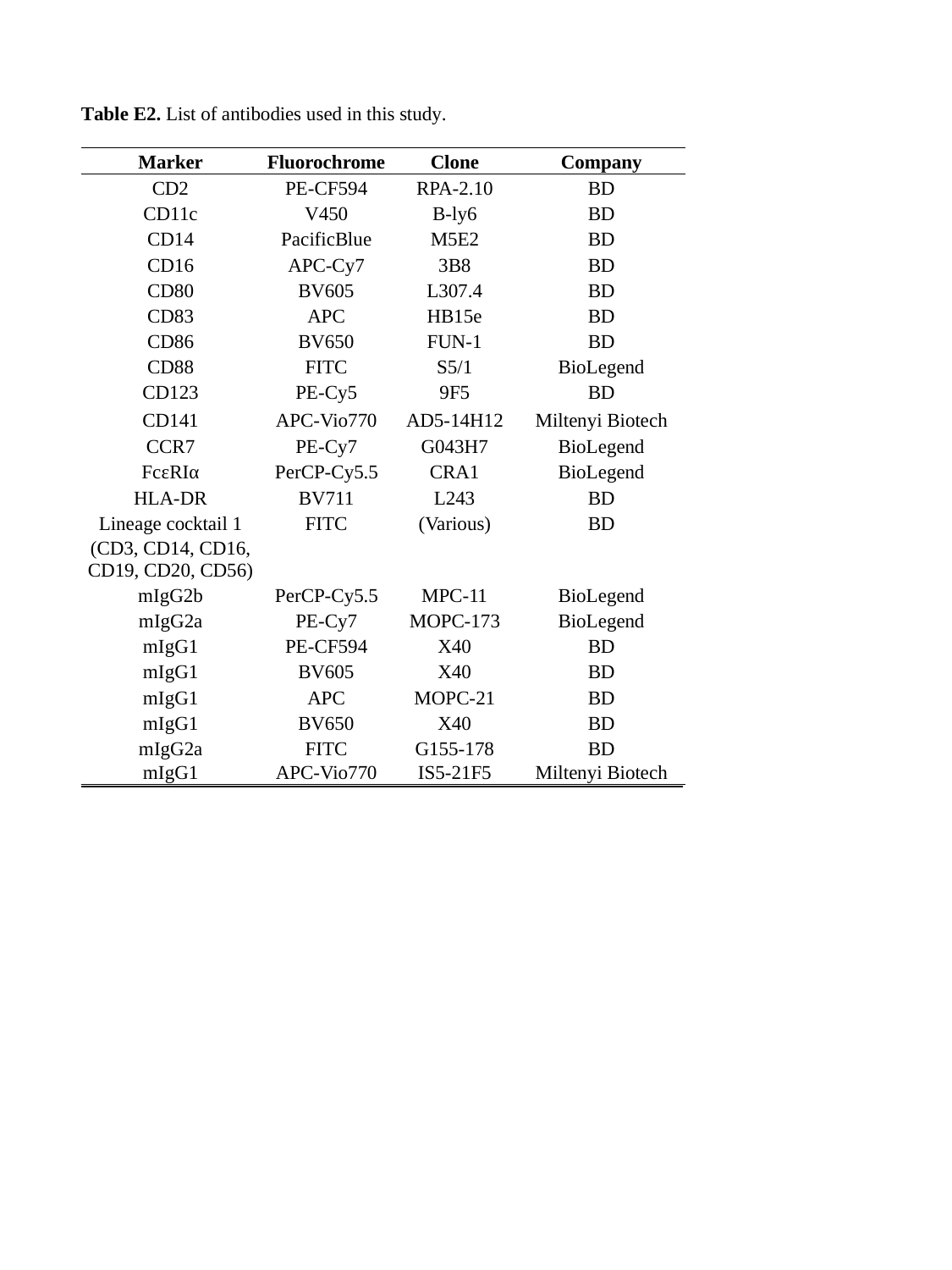

Supplement: Supplementary Tables 1-2 [file mmc2.pptx]
